# Performance evaluation of mainstream large language models in cataract science popularization Q&A: a comparative study of readability, quality, and educational suitability

**DOI:** 10.3389/fopht.2026.1943376

**Published:** 2026-09-24

**Authors:** Yunqing Hou, Mingyue Liu, Zhuowei Wang, Xiaohui Jiang

**Affiliations:** 1 Eye Hospital and School of Ophthalmology and Optometry, Wenzhou Medical University, Wenzhou, China; 2 National Clinical Research Centre for Ocular Diseases, Wenzhou, China

**Keywords:** cataract, digital health, large language models, medical question answering, patient education

## Abstract

**Background:**

Cataract is one of the main causes of visual impairment and reversible blindness worldwide, and it mainly affects the elderly population. Clinically accurate and sufficiently readable patient education materials play a crucial role in this regard. With the rapid development of large language models (LLMs), patients are increasingly obtaining health information generated by artificial intelligence (AI); however, the reliability of online medical information is often questionable. This study systematically evaluated the readability, quality, and educational suitability of mainstream LLMs when answering questions related to cataract.

**Methods:**

Five mainstream LLMs - Doubao, DeepSeek, Wenxin Yiyan, Tongyi Qianwen, and GPT-5 - were evaluated based on their responses to 20 frequently asked questions (FAQs) for cataract patient, which covered five subject categories. Text readability was assessed through multiple indicators, including the Coleman-Liau Index (CL), Linsear Write (LW), Automated Readability Index (ARI), Simple Measure of Gobbledygook (SMOG), Gunning Fog Index (GFOG), Flesch Reading Ease Score (FRES), and Flesch–Kincaid Grade Level (FKGL). Information quality and educational suitability were evaluated using the Global Quality Score (GQS) and the Chinese version of the Patient Education Material Readability Assessment Tool (c-PEMAT-P). Differences between groups were compared using one-way ANOVA and Kruskal-Wallis tests, with correlation analyses exploring relationships among indicators.

**Results:**

There were significant differences among LLMs in terms of readability, information quality, and educational suitability (all p < 0.05). GPT-5 had the highest c-PEMAT-P and GQS scores, however, several readability difficulty indices of GPT-5 and Tongyi Qianwen were also higher. There were significant differences in readability among different content categories. The postoperative management and risk/prevention topics tended to have better educational suitability, whereas surgical diagnosis, treatment, and preoperative management were more difficult to read. Correlation analysis demonstrated that the correlation between the quality indicators and the readability-related indicators is generally weak.

**Conclusion:**

In terms of generating educational materials for cataract patient, LLMs have potential, but the output quality varies depending on the model and the topic. GPT-5 performs best in terms of overall quality and educational suitability, but its readability is not always at an ideal level. Model selection has a crucial impact on the quality and educational suitability of the information, while the content topic mainly affects the language complexity. Therefore, for the responsible use of LLMs in cataract patient education, the cataract education materials generated by LLMs need to undergo expert review, readability optimization, and patient-centered validation before clinical application.

## Introduction

Cataract remains one of the leading causes of visual impairment and reversible blindness worldwide, with the greatest burden concentrated in aging populations (1). As the prevalence of age-related cataract increases, patients and the general public increasingly seek health information on disease prevention, early screening, surgical indications, perioperative preparation, intraocular lens selection, and postoperative follow-up (2, 3). Anxiety levels tend to be high in ophthalmic surgery, especially cataract surgery, often stemming from fear of blindness or surgical failure (4). In this context, patient education materials must not only be clinically accurate but also be sufficiently accessible to support understanding and informed decision making (5).

LLMs are increasingly being used by the public as consumer-facing tools for obtaining health information (6–8). Their ability to generate fluent, structured, and topic-specific responses suggests considerable potential for ophthalmic patient education (9). However, the deployment of LLM-generated content in clinical communication requires careful validation. In particular, the relationship between content quality and readability remains uncertain. A text may be judged by experts as reliable and useful while still requiring a relatively high reading burden for lay readers (10). Conversely, a text that is easier to read may omit clinically relevant detail or fail to provide actionable guidance (11, 12). These two dimensions are therefore related but not interchangeable.

This distinction is especially important in cataract education. The intended audience often includes older adults, patients with variable educational backgrounds, and individuals who may be using Chinese-language consumer LLMs as a first source of information (13, 14). Existing health communication research has repeatedly shown that online patient materials often exceed recommended reading levels, limiting their practical value despite their availability (15). Whether contemporary LLMs can generate Mandarin Chinese cataract education materials that are simultaneously high in quality and accessible in readability remains insufficiently studied.

While several studies have compared LLMs in various medical domains, the following gaps remain: (1) no study has systematically compared the performance of mainstream Chinese domestic LLMs (Doubao, DeepSeek, Wenxin Yiyan, Tongyi Qianwen) alongside an international benchmark (GPT-5) in generating Mandarin Chinese ophthalmic patient education; (2) existing comparisons often focus on a single dimension (e.g., quality or readability), whereas we simultaneously evaluate quality/educational suitability and readability, examining their inter-relationship; and (3) no prior work has assessed whether the performance of LLMs varies across different thematic content categories (e.g., prevention vs. surgical management) within a single disease domain. Accordingly, this study benchmarked these five widely used LLMs, using standardized Mandarin Chinese cataract FAQs for general readers and patients. Output quality was evaluated with c-PEMAT-P and GQS, and readability was assessed using established indices. Under identical prompts, we compared model differences in quality and readability and examined whether these two dimensions were related or independent.

## Methods

### Ethical considerations

The data analyzed in this study were generated entirely by large language models and did not involve any human participants, animal experiments, biological samples, or personally identifiable information. Therefore, ethical review was not required.

### Research procedure

To evaluate the performance of LLMs in cataract knowledge dissemination, two clinical experts developed 20 cataract-related FAQs on June 11, 2026. These FAQs were developed through a multi-step process: (1) a review of the most frequently asked questions encountered in cataract outpatient consultations; (2) reference to the American Academy of Ophthalmology’s Preferred Practice Pattern for Cataract and national Chinese cataract guidelines; and (3) analysis of common patient search queries from a tertiary eye hospital’s online consultation record. The questions were then categorized into five thematic groups (Risk and Prevention, Early Screening, Preoperative Management, Surgical Diagnosis and Treatment, and Postoperative Management) to ensure systematic coverage of the entire cataract care pathway. The full set is provided in **Table 1**.

**Table 1:** Issue list.

Issue list
I. Risk and Prevention 1. Why are people over 50 at high risk of cataracts? 2. Does long-term UV exposure increase cataract risk? 3. How many times higher is cataract risk in diabetic patients? 4. Which common drugs may raise the risk of cataracts? II. Early Screening 1. What are typical early visual symptoms of cataracts? 2. How often should asymptomatic seniors receive eye screening? 3. What is the role of slit-lamp examination in cataract screening? 4. Do diabetic patients need more frequent cataract screening? III. Preoperative Management 1. What general indicators require key assessment before cataract surgery? 2. How to control inflammation for cataracts combined with uveitis? 3. How to use topical antibiotics before routine cataract surgery? 4. Is fundus examination required for cataracts with high myopia? IV. Surgical Diagnosis and Treatment 1. What are the general core indications for cataract surgery? 2. What is the optimal surgical timing for infantile congenital cataracts? 3. Which intraocular lens is the first choice for diabetic patients? 4. Who are suitable for femtosecond laser-assisted cataract surgery? V. Postoperative Management 1. What are the standard follow-up time points after routine cataract surgery? 2. How to treat posterior capsule opacification after cataract surgery? 3. What risky eye behaviors should patients avoid postoperatively? 4. What needs key monitoring for patients with combined glaucoma?

The FAQs were used to query five publicly available LLMs: Doubao, DeepSeek, Wenxin Yiyan, Tongyi Qianwen, and GPT-5 on June 11, 2026. The responses were evaluated for readability, quality, and educational suitability. To balance the breadth of model comparison with the feasibility of manual expert evaluation, one response was collected per question per model. This approach is consistent with previous LLM benchmarking studies in healthcare (16). Because the study used a fixed set of 20 FAQs and was conducted in Chinese, the findings may not fully generalize to other prompt types or multilingual settings.

### LLM query protocol and generation configs

To enhance the repeatability of the benchmark process, we unified the query workflow across all five LLMs. The same prompt template was used for each model for the 20 common questions, and each question was submitted separately (i.e., a new chat session was started with each question) to avoid context effects. The prompt template specifies the target audience (general public/patients), the desired output language (Mandarin Chinese), and the basic formatting requirements (e.g., use plain language, structured bullet points/headings, and avoid unnecessary technical terms).

When the platform allows users to control the generation parameters (such as temperature, top-p, maximum output length/token count), these parameters are set consistently across all models and ensure that the same settings are applied within each model. To address potential model updates over time, each FAQ is analyzed for one model response (20 FAQs × 5 models = 100 outputs). Each output is exported in plain text format for subsequent quality assessment (c-PEMAT-P and GQS) and readability analysis.

### Evaluation of readability

Readability analysis of LLM responses employs a variety of formulas derived from text readability assessment tools. Given that there is no authoritative gold standard or consensus to determine the most reliable measure, the mainstream system widely documented in the literature was therefore used in this study (17).

Readability was evaluated using seven established indices: the CL, LW, ARI, SMOG, GFOG, FRES, and FKGL (18). All outputs were in Mandarin and were not translated at the time of analysis. As these readability formulas were originally developed for English, we mainly used them for relative comparison of text complexity across models/topics rather than for absolute level interpretation in Chinese; Although originally developed for English, these indices have been applied in previous comparative studies of Chinese health information to provide a standardized benchmark for relative text complexity across different sources (19). We acknowledge that absolute scores may not precisely reflect Chinese reading difficulty; however, for the purpose of cross-model and cross-topic comparisons, these metrics provide a useful and consistent yardstick. This was complemented by the c-PEMAT-P for linguistically appropriate assessment. This study was not externally fact-checked by comparing the generated content with current clinical guidelines or other authoritative sources, which may have strengthened the robustness of the findings.

### Quality assessment

Literature quality and accessibility were assessed using two tools: the c-PEMAT-P and the GQS. The c-PEMAT-P includes 24 indicators in two dimensions: intelligibility (16 items, focusing on logical structure and accessibility of terms) and practicability (8 items, assessing specific action guidance and audience appropriateness). Ratings are binary (0/1), so the total score ranges from 0 to 24, with higher values indicating greater user accessibility. In the analysis, we calculated the c-PEMAT-P for each output as a total score from 0 to 24 and then summarized the mean (± SD) of each output within each model/category as a non-integer mean. The GQS uses a 1-5 scale ranging from “poor quality” (1 point: the content is illogical and has no practical value) to “excellent quality” (5 points: logical rigor and significant practical value). Evaluation was performed by 2 independent clinical experts with more than 3 years of relevant clinical experience. This study did not include a real-world comprehension test or patient validation, but it did not directly assess health literacy from a patient’s perspective. To assess inter-rater reliability, Cohen’s kappa coefficient was calculated for the GQS scores, and the intraclass correlation coefficient (ICC) was calculated for the c-PEMAT-P total scores using a two-way random effects model. Values > 0.75 were considered indicative of excellent agreement. The evaluators were blinded to the identity of the LLM that generated each response.

### Statistical analysis

The statistical strategy followed the reference article. Continuous variables were summarized as mean ± standard deviation or median with interquartile range depending on distributional characteristics. Normality was assessed using the Shapiro-Wilk test separately for each model group (n=20 per group), and homogeneity of variance across groups was assessed using Levene’s test. To account for the inherent correlation between responses generated by different models for the same question (i.e., the paired/repeated-measures structure of the data), we employed linear mixed-effects models (LMM) for outcomes meeting normality assumptions, with Model as a fixed effect and Question ID as a random intercept to absorb question-level clustering. For outcomes violating normality assumptions (FRES, FKGL, SMOG, c-PEMAT-P score, and GQS score; see **Table 1**), the Friedman test was used instead. For outcomes meeting normality assumptions, repeated-measures ANOVA was used, with Greenhouse-Geisser correction applied when sphericity was violated. For *post hoc* pairwise comparisons following a significant repeated-measures ANOVA, paired t-tests were used with Bonferroni correction (corrected α = 0.005 for 10 pairwise comparisons). For *post hoc* comparisons following a significant Friedman test, Wilcoxon signed-rank tests were used with Bonferroni correction. Correlations between quality metrics and readability indices were assessed using Spearman’s rank correlation coefficient, given the non-normal distribution of several variables. All tests were two-sided, and a p value less than 0.05 was considered statistically significant.

## Results

### Readability analysis

This study systematically evaluated the impact of different LLMs and different health education dimensions on the readability, educational suitability, and information quality of educational materials for cataract patients. At the model level, c-PEMAT-P, GQS, and multiple readability indicators (ARI, FRES, GFOG, FKGL, CL, SMOG, and LW) were compared on five commonly used LLMs (Doubao, DeepSeek, Wenxin Yiyan, Tongyi Qianwen, and GPT-5). At the content level, these indicators were further analyzed for five education dimensions related to cataracts (Risk and Prevention, Early Screening, Preoperative Management, Surgical Diagnosis and Treatment, and Postoperative Management).

In terms of the educational suitability (**Table 2**), the text generated by GPT-5 received the highest c-PEMAT-P score (12.95 ± 0.83), followed by Doubao (11.75 ± 1.68) and Tongyi Qianwen (11.75 ± 1.62). Wenxin Yiyan performed moderately (11.60 ± 1.39), while DeepSeek scored the lowest (11.45 ± 1.32). The Friedman test revealed significant differences among the models (χ² = 18.22, p = 0.001). *Post hoc* comparisons with Bonferroni correction (corrected α = 0.005) showed that GPT-5 scored significantly higher than Wenxin Yiyan (p = 0.005), but the difference between GPT-5 and DeepSeek did not reach the adjusted significance threshold (p = 0.007) (**Supplementary Table 2**). The overall quality evaluated by GQS also showed a similar trend, with GPT-5 scoring the highest (4.15 ± 0.67), followed by DeepSeek (3.00 ± 1.03) and Doubao (2.90 ± 1.12). The GQS scores of Tongyi Qianwen and Wenxin Yiyan were lower (2.15 ± 0.99 and 2.15 ± 0.93, respectively). The Friedman test demonstrated significant differences among models (χ² = 37.69, p < 0.001). *Post hoc* comparisons showed that GPT-5 scored significantly higher than Tongyi Qianwen (p = 0.002) and Wenxin Yiyan (p = 0.001) (**Supplementary Table 2**). These results indicate that GPT-5 outperforms the other models in terms of the content structure, information organization, and overall quality of cataract patient education materials.

**Table 2:** Comparison of readability and quality indicators across five LLMs.

Variables	Total (n = 100)	Deep Seek (n = 20)	Doubao (n = 20)	GPT-5 (n = 20)	Tongyi Qianwen (n = 20)	Wenxin Yiyan (n = 20)	Statistic	P	P (GG correction)
ARI (M,Q1,Q3)	17.58(15.88,18.98)	16.43(15.47,18.02)	15.90(14.42,17.72)	17.93(16.98,18.58)	20.04(18.69,21.27)	16.92(15.88,18.35)	F=22.29	<.001	<.001(GG)
FRES (M,Q1,Q3)	11.00(0.00,24.00)	16.50(0.00,27.00)	21.00(11.25,31.25)	10.00(0.00,20.00)	0.00(0.00,3.00)	15.50(6.75,24.25)	χ²=33.44	<.001	—
GFOG (M,Q1,Q3)	18.10(15.78,20.07)	17.10(15.60,20.90)	16.10(13.73,18.82)	18.10(16.03,19.27)	22.15(19.23,23.75)	17.10(16.38,18.77)	F=26.15	<.001	<.001(GG)
FKGL (M,Q1,Q3)	16.16(14.61,17.96)	15.51(14.20,17.08)	15.04(13.43,16.71)	17.19(15.16,18.01)	19.20(17.64,20.16)	15.71(14.46,16.98)	χ²=42.32	<.001	—
CL (M,Q1,Q3)	18.09(16.55,20.48)	17.76(16.35,20.41)	16.92(14.89,18.24)	18.01(16.87,19.54)	22.27(20.22,24.16)	17.38(16.53,19.59)	F=37.07	<.001	<.001(GG)
SMOG (M,Q1,Q3)	14.03(12.56,15.27)	13.24(12.79,14.33)	12.57(12.09,14.36)	14.80(13.19,15.67)	15.54(14.97,16.30)	13.95(13.05,14.36)	χ²=35.95	<.001	—
LW (M,Q1,Q3)	53.00(49.00,58.00)	53.50(50.75,58.25)	56.50(52.75,59.25)	50.00(49.00,54.00)	48.50(44.50,53.00)	56.00(51.75,60.00)	F=10.85	<.001	<.001(GG)
C-PEMAT score (Mean ± SD)	11.90 ± 1.47	11.45 ± 1.32	11.75 ± 1.68	12.95 ± 0.83	11.75 ± 1.62	11.60 ± 1.39	χ²=18.22	0.001	—
GQS score (Mean ± SD)	2.87 ± 1.19	3.00 ± 1.03	2.90 ± 1.12	4.15 ± 0.67	2.15 ± 0.99	2.15 ± 0.93	χ²=37.69	<.001	—

F statistics are from repeated-measures ANOVA with Platform as the within-subjects factor and Question ID as the blocking factor; χ² statistics are from the Friedman test (df = 4). The GG-corrected P values, shown in the last column, were calculated using the Greenhouse–Geisser correction for sphericity and are reported only for the four indicators analyzed with repeated-measures ANOVA.

SD, standard deviation; M, Median; Q₁, 1st Quartile; Q₃, 3rd Quartile.

In terms of readability-related indicators (ARI, FRES, GFOG, FKGL, CL, SMOG, and LW), significant differences were also observed among models (all p < 0.001; **Table 2**). Tongyi Qianwen and GPT-5 scored higher on ARI, GFOG, FKGL, CL, and SMOG, indicating higher language complexity and greater reading demands. In contrast, Doubao scored lower on these indicators, suggesting a relatively lighter reading burden. For ARI, repeated-measures ANOVA revealed a significant model effect (F = 22.29, p < 0.001). *Post hoc* paired t-tests with Bonferroni correction showed that Tongyi Qianwen had significantly higher ARI scores than all other models (all p < 0.001), and GPT-5 scored significantly higher than Doubao (p < 0.001) and DeepSeek (p < 0.001). For the FRES indicator, Doubao had the highest score, indicating easier readability, while Tongyi Qianwen had the lowest. The Friedman test showed significant differences (χ² = 33.44, p < 0.001), with *post hoc* tests confirming that Doubao scored significantly higher than Tongyi Qianwen (p = 0.003). For the LW indicator, Doubao showed the highest proportion of long words, while Tongyi Qianwen and GPT-5 had relatively lower values; repeated-measures ANOVA showed significant differences among models (F = 10.85, p < 0.001).

The content-level analysis (**Table 3**; **Supplementary Table 3**) shows that there were significant differences in text readability across content dimensions for most readability indicators (ARI, FRES, GFOG, FKGL, CL, SMOG, and LW; all p < 0.05). Texts involving Preoperative Management and Surgical Diagnosis and Treatment require a higher reading level, indicating a higher language complexity. However, when employing linear mixed-effects models with Question ID as a random intercept to account for the paired data structure, the differences in c-PEMAT-P scores across content dimensions were not statistically significant (F = 1.22, p = 0.344), nor were the differences in GQS scores (F = 1.33, p = 0.303).

**Table 3:** Comparison of readability and quality indicators across five content dimensions.

Variables	Total (n = 100)	Early screening (n = 20)	Postoperative management (n = 20)	Preoperative management (n = 20)	Risk and prevention (n = 20)	Surgical diagnosis and treatment (n = 20)	F (dimension)	P
ARI (M,Q1,Q3)	17.58 (15.88,18.98)	16.59 (15.67,18.38)	16.94 (14.00,17.75)	18.38 (17.53,20.50)	16.24 (15.53,17.40)	18.73 (18.08,19.49)	F=3.36	0.037
FRES (M,Q1,Q3)	11.00 (0.00,24.00)	16.50 (6.00,29.00)	23.50 (16.25,36.25)	0.00 (0.00,7.25)	18.00 (14.25,28.50)	1.50 (0.00,9.75)	F=4.80	0.011
GFOG (M,Q1,Q3)	18.10 (15.78,20.07)	17.40 (13.73,19.07)	16.10 (13.07,16.50)	20.70 (18.75,22.43)	16.10 (15.45,18.55)	19.35 (18.35,21.20)	F=6.04	0.004
FKGL (M,Q1,Q3)	16.16 (14.61,17.96)	15.60 (14.11,17.62)	15.04 (12.35,15.75)	17.59 (16.82,19.78)	15.10 (14.37,15.67)	17.80 (17.03,19.46)	F=5.43	0.007
CL (M,Q1,Q3)	18.09 (16.55,20.48)	17.15 (15.75,19.50)	16.76 (15.52,18.64)	20.75 (19.38,22.36)	17.58 (16.32,19.02)	19.45 (17.89,21.52)	F=4.26	0.017
SMOG (M,Q1,Q3)	14.03 (12.56,15.27)	14.29 (11.57,15.11)	12.54 (10.69,14.11)	14.49 (13.32,15.96)	13.16 (12.43,13.81)	15.65 (14.88,16.26)	F=3.43	0.035
LW (M,Q1,Q3)	53.00 (49.00,58.00)	53.50 (49.00,57.25)	57.50 (50.00,60.50)	51.50 (46.50,54.00)	60.00 (53.75,61.00)	49.00 (47.00,52.25)	F=4.19	0.018
C-PEMAT score (Mean ± SD)	11.90 ± 1.47	11.25 ± 1.74	12.45 ± 1.19	12.00 ± 1.59	12.35 ± 1.23	11.45 ± 1.28	F=1.22	0.344
GQS score (Mean ± SD)	2.87 ± 1.19	2.90 ± 1.21	2.95 ± 1.23	2.85 ± 1.31	3.30 ± 0.98	2.35 ± 1.14	F=1.33	0.303

Linear mixed-effects models were used with Dimension and Platform as fixed effects and Question ID as a random intercept (to account for question-level clustering). REML estimation was applied with Satterthwaite approximation for denominator degrees of freedom.

SD, standard deviation; M, Median; Q₁, 1st Quartile; Q₃, 3rd Quartile.

### Quality analysis

As shown in **Figure 1**, the distribution of GQS scores for each model reveals significant differences in overall text quality. Compared to the other four models, GPT-5 achieved the highest GQS score, with the median approaching the upper limit of the scale (5.0), and the interquartile range being the narrowest. The text quality generated by this model is highly concentrated, reflecting strong stability and consistency. Doubao and DeepSeek had moderate scores, and there was no statistically significant difference between the two models (ns). Compared to GPT-5, their interquartile range was slightly wider, indicating a slight reduction in consistency. In contrast, Wenxin Yiyan and Tongyi Qianwen had significantly lower GQS scores, all below 3. One-way ANOVA confirmed that there were significant differences in GQS scores among different platforms (p < 0.001). According to the standard GQS scoring criteria, a score of 4-5 indicates high-quality information, while a score of 3 represents medium quality. The corresponding violin plot shows a wider distribution and a distinct long-tail feature, indicating greater differences in text quality and a higher proportion of low-quality outputs.

**Figure 1:**
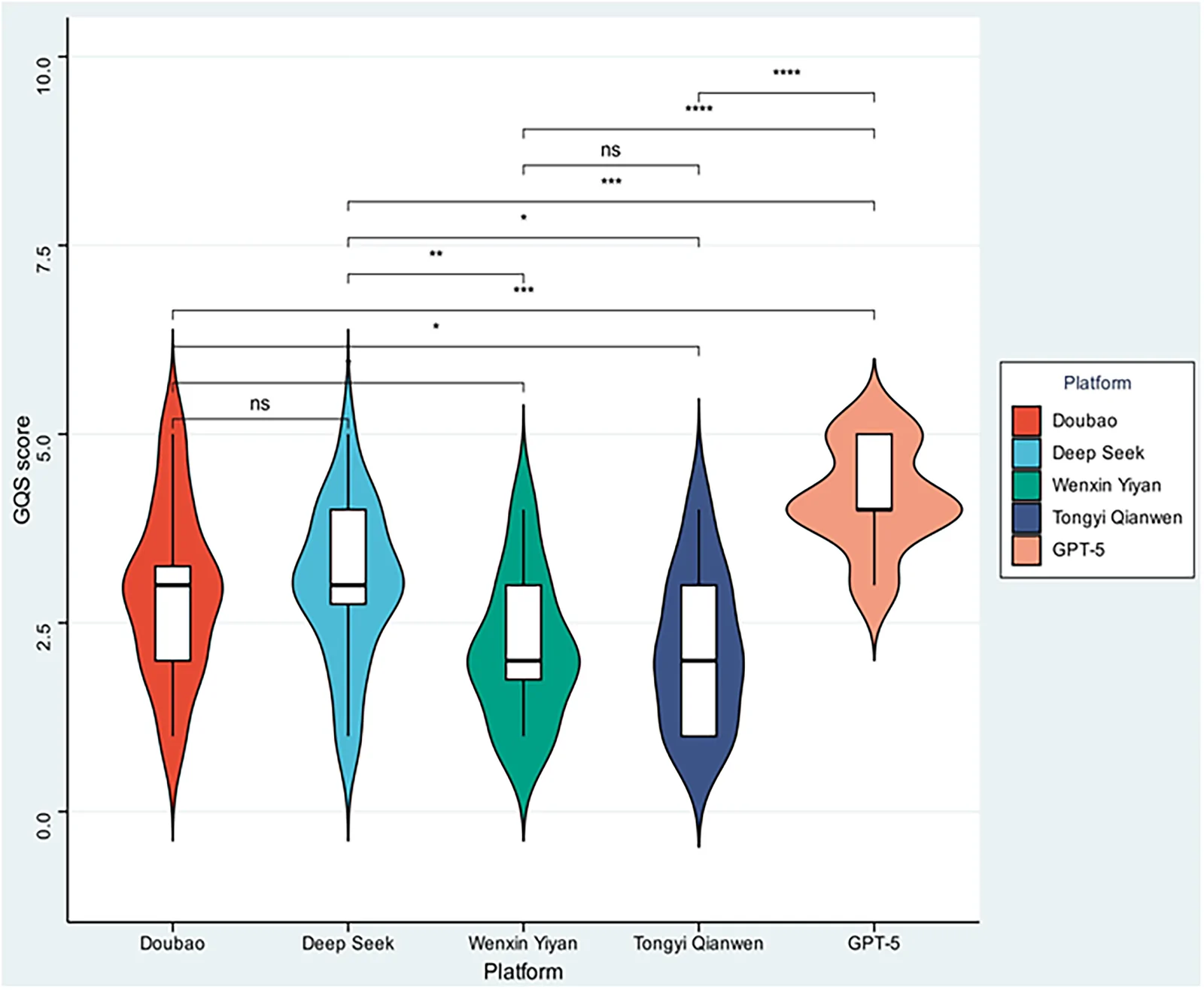
GQS scores of different mainstream large language models. Comparison of GQS scores across five mainstream large language models. Violin plots show the distribution of GQS scores for Doubao, Deep Seek, Wenxin Yiyan, Tongyi Qianwen, and GPT-5. The embedded boxplots indicate the median and interquartile range. Pairwise comparisons between platforms are shown above the plots, with significance levels indicated by asterisks and “ns” denoting non-significant differences. *:p < 0.05 **:p < 0.01 ***:p < 0.001 ****:p < 0.0001.

Within the **Figure 2**, the c-PEMAT-P scores of each model are mostly between 11 and 13, indicating that the generated health education materials as a whole have good comprehensibility and operability. Paired comparisons show that the differences between most models are not statistically significant, but there are significant differences between certain specific model combinations (p < 0.001). It is worth noting that the median score of GPT-5 is the highest, and the interquartile range is the narrowest, reflecting its most stable performance in generating education content suitable for patients. In contrast, the scores of Doubao and Tongyi Qianwen were the lowest and their distribution was the most scattered. This indicates that the generated texts by these two models may have problems such as excessive use of technical terms or unclear guidance, which is not conducive to patients’ understanding and implementation of health knowledge. There are significant differences in the applicability of the educational content. The inter-rater reliability was substantial for both instruments, with Cohen’s κ = 0.82 (95% CI: 0.74–0.90) for GQS and ICC = 0.79 (95% CI: 0.70–0.86) for c-PEMAT-P, indicating a high level of agreement between the two evaluators. Disagreements were resolved through consensus discussion.

**Figure 2:**
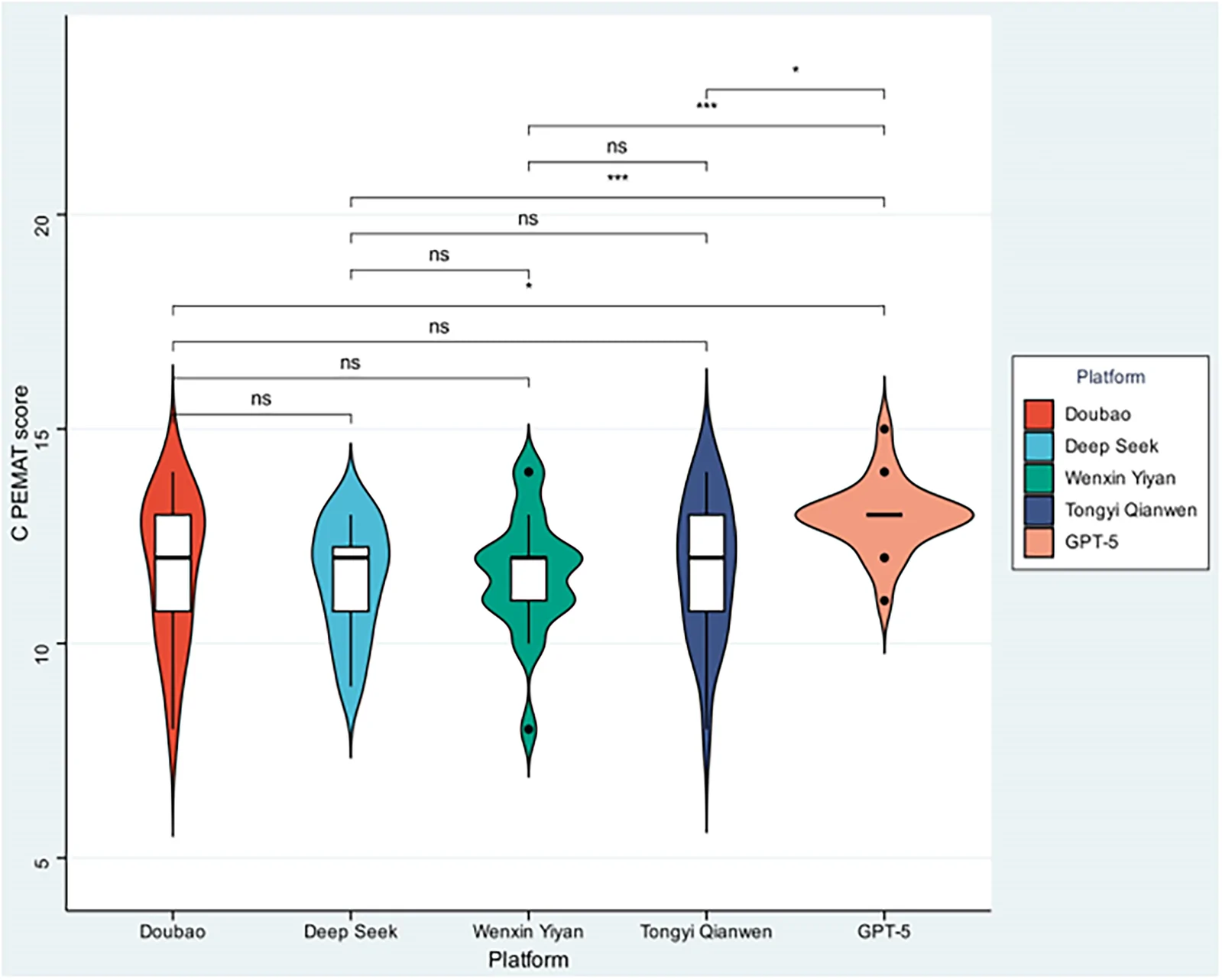
c-PEMAT-P scores of different mainstream large language models. Comparison of c-PEMAT-P scores across five mainstream large language models. Violin plots show the distribution of c-PEMAT-P scores for Doubao, Deep Seek, Wenxin Yiyan, Tongyi Qianwen, and GPT-5. The embedded boxplots indicate the median and interquartile range. Pairwise comparisons between platforms are shown above the plots, with significance levels indicated by asterisks and “ns” denoting non-significant differences. *:p < 0.05 **:p < 0.01 ***:p < 0.001 ****:p < 0.0001.

### Correlation analysis

The correlation distribution in the heatmap (**Figure 3**) reveals that the weak correlations between quality metrics (GQS, c-PEMAT-P) and readability indices, as assessed by Spearman’s rank correlation coefficient, confirm that these two dimensions—while partially overlapping—are largely independent constructs in the context of AI-generated patient education. This suggests that a text can be rated as high-quality by expert evaluators while remaining difficult to read for lay audiences, and conversely, an easier-to-read text may not necessarily be of higher quality.

**Figure 3:**
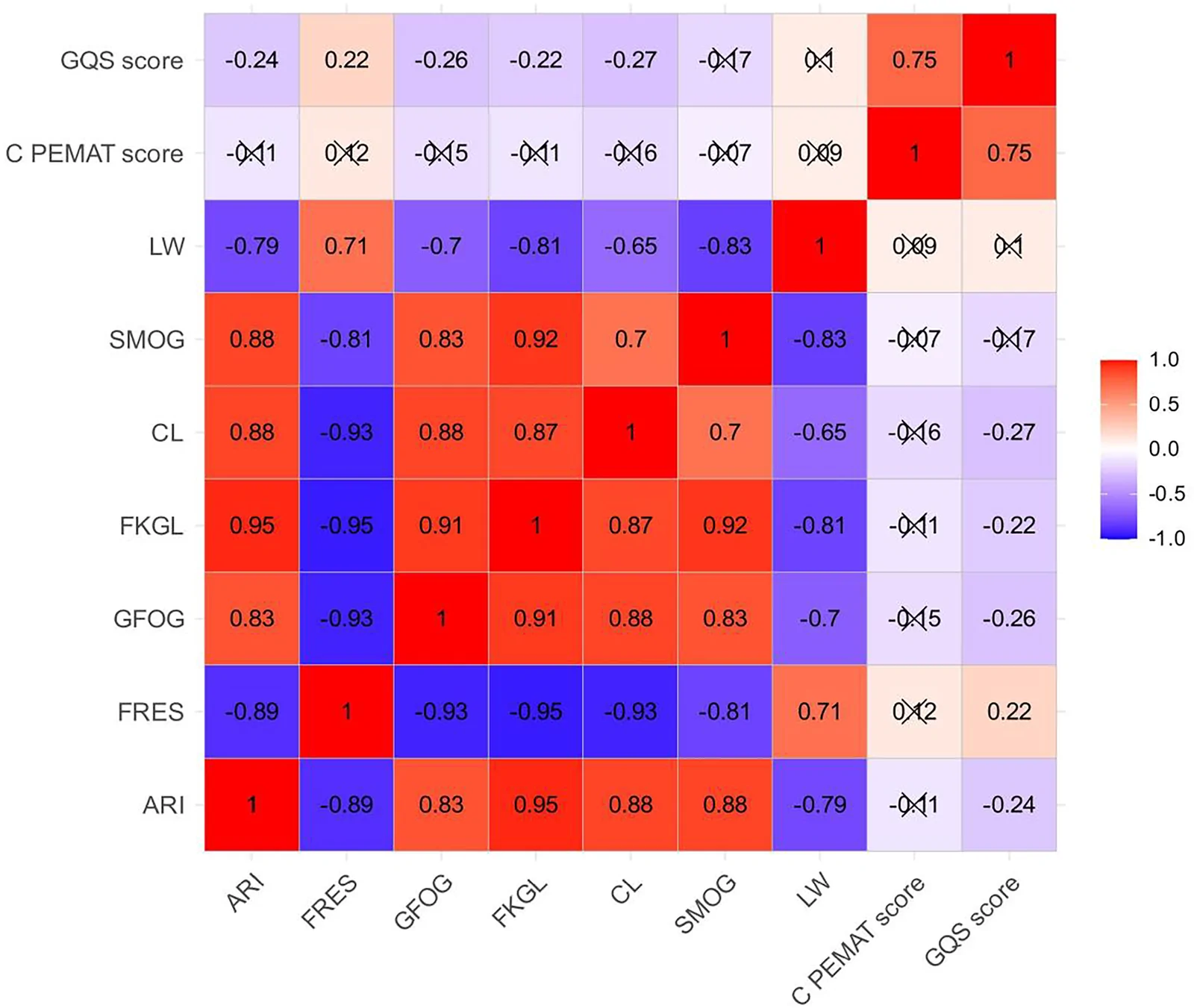
Heatmap of correlations among readability and quality assessment metrics. Correlation heatmap of readability and quality assessment metrics. The heatmap shows correlation coefficients among readability indices and quality assessment metrics, including ARI, FRES, GFOG, FKGL, CL, SMOG, LW, c-PEMAT-P score and GQS score. Red indicates positive correlation and blue indicates negative correlation. Black crosses mark non-significant correlations.

In the quality dimension, GQS has a weak positive correlation with FRES (ρ = 0.22), suggesting that texts with higher quality scores tend to be more readable. Additionally, GQS has a weak negative correlation with ARI (ρ = -0.24), GFOG (ρ = -0.26), and CL (ρ = -0.27), indicating that texts with higher quality scores are more suitable for patients with lower educational levels. In contrast, the c-PEMAT-P score has a very weak correlation with the traditional readability-related indicators. The correlation coefficients between c-PEMAT-P and ARI, FRES, GFOG, FKGL, CL, SMOG, and LW are close to zero (all |ρ| < 0.2), suggesting that the suitability of patient education is largely unaffected by sentence complexity and vocabulary difficulty.

A further analysis of the internal structure of the readability-related indicators reveals a strong and consistent correlation among them. For instance, there is a significant positive correlation between ARI and FKGL (ρ = 0.95), and FKGL and SMOG also show a significant positive correlation (ρ = 0.92), while ARI and SMOG also have a significant positive correlation (ρ = 0.88). These further demonstrate that the readability-related indicators can serve as a collaborative assessment tool for “text reading difficulty”, but their direct driving effect on “text quality” is limited. This result indicates that “text quality” and “text reading difficulty” are relatively independent dimensions; quality is more dependent on the accuracy and suitability of the information, while readability is dominated by language complexity (vocabulary proficiency, sentence complexity, text length, etc.).

## Discussion

This study compared five large language models in generating Mandarin Chinese cataract patient education materials. The results showed significant differences among models in educational suitability, overall quality, and readability (20, 21). GPT-5 had the highest c-PEMAT-P and GQS scores, suggesting better performance in content organization, logical coherence, and patient-oriented information delivery. However, several readability difficulty indices of GPT-5 and Tongyi Qianwen were also higher, indicating that their language complexity was higher. In contrast, the text generated by Doubao was relatively easy to read but did not receive the highest quality scores. These findings suggest that higher information quality does not necessarily correspond to better readability, as detailed in other studies (22). Our results add to a growing body of evidence across various medical specialties that comparatively evaluates LLM performance in patient education and clinical question answering, such as in orthopedics and infectious disease (20, 23). There are also studies comparing AI and residents passing specialty exams that show that the ability of LLMs to provide accurate answers declines with increasing question classification and complexity, suggesting gaps in implementation knowledge (24).

The superior performance of GPT-5 may reflect its stronger ability to organize medical information and provide structured explanations. Cataract education involves multiple domains, including risk factors, screening, surgical indications, intraocular lens selection, perioperative preparation, and postoperative care (25, 26). High-quality patient materials should therefore be accurate, coherent, and actionable. The higher GQS and c-PEMAT-P scores of GPT-5 indicate that its responses were more likely to meet these requirements. The dissociation between quality and readability observed in our study—where GPT-5 achieved the highest GQS and c-PEMAT-P scores but also demonstrated relatively high language complexity—deserves particular attention. This dissociation carries important implications: quality assessment and readability assessment should be treated as complementary rather than interchangeable steps in evaluating LLM-generated patient materials. Clinicians and educators should not assume that a high-quality response is automatically suitable for patients with low health literacy. A response that is logically structured, comprehensive, and clinically relevant may still impose a significant reading burden on older adults or those with limited educational backgrounds. Conversely, a response that is easier to read may omit essential clinical details or fail to provide actionable guidance. Therefore, evaluating AI-generated patient education materials requires a dual-axis framework that simultaneously considers both content quality (what is said) and readability (how it is said). This distinction is especially critical in ophthalmology, where the target population often includes elderly patients with age-related declines in visual function and cognitive processing, further compounding the challenges of health information comprehension. Nevertheless, the overall c-PEMAT-P scores remained moderate, suggesting that even high-performing LLMs still have limitations in generating fully patient-accessible materials. This is particularly important because cataract patients are often older adults with variable health literacy and visual function (27). In a cross-sectional study assessing the ability of an artificial intelligence chatbot assistant (ChatGPT) to provide high-quality and empathic responses to patient questions, chatbots produced high-quality and empathic responses to patients’ questions in an online forum, which may also suggest a better fit for older adults without health literacy, which is also an advantage of AI, Further studies are warranted in the future (28).

At the content level, postoperative management and risk/prevention topics tended to have better educational suitability, whereas surgical diagnosis, treatment, and preoperative management were more difficult to read. This topic-dependent variability in LLM-generated patient education materials has also been observed in other disease contexts. For instance, in patient education for foot and ankle disorders, the complexity and actionability of AI-generated content were similarly found to vary across different clinical topics (29). This may be because prevention and postoperative care can be expressed as concrete behavioral instructions, while surgical topics require more technical explanations (30, 31). These results suggest that topic-specific prompt optimization may be needed. For complex cataract-related topics, LLMs should be guided to use shorter sentences, explain medical terms, and provide clearer action-oriented recommendations.

Several limitations should be acknowledged. First, the readability formulas used are primarily validated for English texts. While we used them for relative comparisons among models and content categories, their absolute scores may not accurately reflect the reading difficulty of Mandarin Chinese materials. The lack of spaces between Chinese characters and the different syllabic structures may affect formula outputs. Future studies should incorporate readability measures specifically designed and validated for the Chinese language or, ideally, include direct patient comprehension testing.

Second, we generated only one response per question per model. Given the stochastic nature of LLMs and the potential variability across repeated queries with identical prompts, our findings should be interpreted as a snapshot of model performance rather than a definitive ranking. Future work should employ multiple generations per question to assess within-model variability and response consistency, perhaps using metrics such as semantic similarity or answer consistency scores.

Third, while our two expert evaluators assessed the quality and educational suitability of the responses using GQS and c-PEMAT-P, we did not perform an independent, systematic fact-check of each response against current clinical guidelines (e.g., the American Academy of Ophthalmology Preferred Practice Pattern for Cataract). Thus, our findings reflect the models’ ability to organize and present information in a comprehensible and actionable manner, but they do not directly establish clinical accuracy, completeness, or safety. A response could receive a high GQS score while still containing clinically incorrect or incomplete information. Future research should incorporate a separate clinical accuracy assessment by domain experts, ideally using a structured scoring rubric for correctness and potential harm.

Fourth, to standardize the evaluation across all models, we used a fixed set of 20 FAQs. We acknowledge that several questions—such as those regarding intraocular lens selection—are clinically nuanced and may not have a single universally correct answer, often depending on individual patient characteristics (e.g., lifestyle, comorbidities, ocular anatomy). Our objective was to assess how LLMs handle typical patient-oriented queries, rather than to evaluate their performance in complex clinical decision-making. The “correctness” of such responses may be better evaluated using a patient-centered utility framework, which we recommend for future studies.

Finally, and most importantly, our evaluation relied entirely on expert and formula-based assessments. We did not include real cataract patients, many of whom are older adults with heterogeneous levels of health literacy, varying educational backgrounds, and—importantly—visual impairment that may affect their ability to read and comprehend written materials. Actual patient comprehension, perceived usefulness, information recall, and decision-making after reading LLM-generated texts remain unknown. Readability scores derived from formulas cannot substitute for direct usability testing with the target population. Future research must prioritize patient-centered evaluations, including readability testing, comprehension interviews, and usability assessments, before any LLM-generated materials can be recommended for clinical deployment.

In conclusion, LLMs show potential for supporting cataract patient education, but their outputs vary significantly by model and topic. GPT-5 provided the highest overall quality and educational suitability, but its readability was not consistently optimal. Quality and readability should therefore be assessed separately. Before clinical use, LLM-generated cataract education materials require expert review, readability optimization, and patient-centered validation.

## Conclusion

In terms of generating educational materials for cataract patient, LLMs have potential, but the output quality varies depending on the model and the topic. GPT-5 performs best in terms of overall quality and educational suitability under the prompts and experimental conditions evaluated in this study, but its readability is not always at an ideal level. Model selection has a crucial impact on the quality and educational suitability of the information, while the content topic mainly affects the language complexity. Therefore, for the responsible use of LLMs in cataract patient education, the cataract education materials generated by LLMs need to undergo expert review, readability optimization, and patient-centered validation before clinical application.

## Data Availability

The raw data supporting the conclusions of this article will be made available by the authors, without undue reservation.
