# Supplementary material for: Performance evaluation of mainstream large language models in cataract science popularization Q&A: a comparative study of readability, quality, and educational suitability
Source: Front Ophthalmol (Lausanne). 2026 Sep 24;6:1943376. doi: 10.3389/fopht.2026.1943376 (PMC13645593; doi:10.3389/fopht.2026.1943376)
Supplement: Supplementary file 1 [file Table1.docx]

**Supplementary Table 1.**

**Normality and homoscedasticity assessment for each indicator**

**(Shapiro–Wilk / Levene’s test, per platform n = 20)**

| Variables | Shapiro–Wilk W range | P range | Platforms rejecting normality (n/5) | Levene’s F | Levene’s P | Test method adopted |
| --- | --- | --- | --- | --- | --- | --- |
| ARI | 0.917–0.979 | 0.087–0.920 | 0/5 | 0.498 | 0.737 | Repeated-measures ANOVA |
| FRES | 0.566–0.948 | 0.000–0.345 | 3/5 | 1.788 | 0.138 | Friedman test |
| GFOG | 0.916–0.971 | 0.084–0.783 | 0/5 | 1.344 | 0.259 | Repeated-measures ANOVA |
| FKGL | 0.884–0.978 | 0.021–0.910 | 1/5 | 0.127 | 0.972 | Friedman test |
| CL | 0.928–0.984 | 0.144–0.974 | 0/5 | 0.426 | 0.790 | Repeated-measures ANOVA |
| SMOG | 0.879–0.980 | 0.017–0.930 | 3/5 | 0.085 | 0.987 | Friedman test |
| LW | 0.931–0.955 | 0.163–0.457 | 0/5 | 0.391 | 0.815 | Repeated-measures ANOVA |
| C-PEMAT score | 0.819–0.935 | 0.002–0.197 | 2/5 | 3.888 | 0.006 | Friedman test |
| GQS score | 0.798–0.921 | 0.001–0.102 | 3/5 | 0.816 | 0.518 | Friedman test |

Note: Shapiro–Wilk tests were performed separately for each platform group (n = 20) for each outcome variable. Variables with all five platforms showing P ≥ 0.05 were analyzed using repeated-measures ANOVA; variables with any platform showing P < 0.05 were analyzed using the Friedman test. Homoscedasticity was assessed using Levene’s test based on the mean (grouped by platform, df₁ = 4, df₂ = 95). Except for c-PEMAT-P score, all indicators met the homogeneity of variance assumption (P ≥ 0.05). The heterogeneity of c-PEMAT-P score (P = 0.006) is consistent with its non-normal distribution and the use of the Friedman test, which does not rely on the homoscedasticity assumption.

**Supplementary Table 2.**

**Pairwise post hoc comparisons between platforms with Bonferroni correction**

| Variables | Comparison | Method | Raw P | Adjusted P | Significant (adj. P < 0.005) |
| --- | --- | --- | --- | --- | --- |
| ARI | Deep Seek vs Doubao | Paired t-test | 0.131 | 1.000 | No |
| ARI | Deep Seek vs GPT-5 | Paired t-test | 0.008 | 0.082 | No |
| ARI | Deep Seek vs Tongyi Qianwen | Paired t-test | <.001 | <.001 | Yes |
| ARI | Deep Seek vs Wenxin Yiyan | Paired t-test | 0.945 | 1.000 | No |
| ARI | Doubao vs GPT-5 | Paired t-test | <.001 | <.001 | Yes |
| ARI | Doubao vs Tongyi Qianwen | Paired t-test | <.001 | <.001 | Yes |
| ARI | Doubao vs Wenxin Yiyan | Paired t-test | 0.247 | 1.000 | No |
| ARI | GPT-5 vs Tongyi Qianwen | Paired t-test | <.001 | <.001 | Yes |
| ARI | GPT-5 vs Wenxin Yiyan | Paired t-test | 0.028 | 0.277 | No |
| ARI | Tongyi Qianwen vs Wenxin Yiyan | Paired t-test | <.001 | <.001 | Yes |
| FRES | Deep Seek vs Doubao | Wilcoxon signed-rank | 0.093 | 0.926 | No |
| FRES | Deep Seek vs GPT-5 | Wilcoxon signed-rank | 0.036 | 0.359 | No |
| FRES | Deep Seek vs Tongyi Qianwen | Wilcoxon signed-rank | <.001 | 0.010 | No |
| FRES | Deep Seek vs Wenxin Yiyan | Wilcoxon signed-rank | 0.965 | 1.000 | No |
| FRES | Doubao vs GPT-5 | Wilcoxon signed-rank | 0.001 | 0.010 | No |
| FRES | Doubao vs Tongyi Qianwen | Wilcoxon signed-rank | <.001 | 0.003 | Yes |
| FRES | Doubao vs Wenxin Yiyan | Wilcoxon signed-rank | 0.211 | 1.000 | No |
| FRES | GPT-5 vs Tongyi Qianwen | Wilcoxon signed-rank | <.001 | 0.010 | No |
| FRES | GPT-5 vs Wenxin Yiyan | Wilcoxon signed-rank | 0.091 | 0.906 | No |
| FRES | Tongyi Qianwen vs Wenxin Yiyan | Wilcoxon signed-rank | <.001 | 0.003 | Yes |
| GFOG | Deep Seek vs Doubao | Paired t-test | 0.020 | 0.201 | No |
| GFOG | Deep Seek vs GPT-5 | Paired t-test | 0.487 | 1.000 | No |
| GFOG | Deep Seek vs Tongyi Qianwen | Paired t-test | <.001 | <.001 | Yes |
| GFOG | Deep Seek vs Wenxin Yiyan | Paired t-test | 0.294 | 1.000 | No |
| GFOG | Doubao vs GPT-5 | Paired t-test | 0.007 | 0.071 | No |
| GFOG | Doubao vs Tongyi Qianwen | Paired t-test | <.001 | <.001 | Yes |
| GFOG | Doubao vs Wenxin Yiyan | Paired t-test | 0.150 | 1.000 | No |
| GFOG | GPT-5 vs Tongyi Qianwen | Paired t-test | <.001 | <.001 | Yes |
| GFOG | GPT-5 vs Wenxin Yiyan | Paired t-test | 0.504 | 1.000 | No |
| GFOG | Tongyi Qianwen vs Wenxin Yiyan | Paired t-test | <.001 | <.001 | Yes |
| FKGL | Deep Seek vs Doubao | Wilcoxon signed-rank | 0.737 | 1.000 | No |
| FKGL | Deep Seek vs GPT-5 | Wilcoxon signed-rank | 0.006 | 0.064 | No |
| FKGL | Deep Seek vs Tongyi Qianwen | Wilcoxon signed-rank | <.001 | 0.001 | Yes |
| FKGL | Deep Seek vs Wenxin Yiyan | Wilcoxon signed-rank | 0.940 | 1.000 | No |
| FKGL | Doubao vs GPT-5 | Wilcoxon signed-rank | <.001 | 0.002 | Yes |
| FKGL | Doubao vs Tongyi Qianwen | Wilcoxon signed-rank | <.001 | <.001 | Yes |
| FKGL | Doubao vs Wenxin Yiyan | Wilcoxon signed-rank | 0.444 | 1.000 | No |
| FKGL | GPT-5 vs Tongyi Qianwen | Wilcoxon signed-rank | <.001 | <.001 | Yes |
| FKGL | GPT-5 vs Wenxin Yiyan | Wilcoxon signed-rank | 0.012 | 0.117 | No |
| FKGL | Tongyi Qianwen vs Wenxin Yiyan | Wilcoxon signed-rank | <.001 | 0.001 | Yes |
| CL | Deep Seek vs Doubao | Paired t-test | 0.015 | 0.149 | No |
| CL | Deep Seek vs GPT-5 | Paired t-test | 0.429 | 1.000 | No |
| CL | Deep Seek vs Tongyi Qianwen | Paired t-test | <.001 | <.001 | Yes |
| CL | Deep Seek vs Wenxin Yiyan | Paired t-test | 0.587 | 1.000 | No |
| CL | Doubao vs GPT-5 | Paired t-test | <.001 | 0.001 | Yes |
| CL | Doubao vs Tongyi Qianwen | Paired t-test | <.001 | <.001 | Yes |
| CL | Doubao vs Wenxin Yiyan | Paired t-test | 0.162 | 1.000 | No |
| CL | GPT-5 vs Tongyi Qianwen | Paired t-test | <.001 | <.001 | Yes |
| CL | GPT-5 vs Wenxin Yiyan | Paired t-test | 0.220 | 1.000 | No |
| CL | Tongyi Qianwen vs Wenxin Yiyan | Paired t-test | <.001 | <.001 | Yes |
| SMOG | Deep Seek vs Doubao | Wilcoxon signed-rank | 0.433 | 1.000 | No |
| SMOG | Deep Seek vs GPT-5 | Wilcoxon signed-rank | 0.014 | 0.137 | No |
| SMOG | Deep Seek vs Tongyi Qianwen | Wilcoxon signed-rank | <.001 | 0.005 | No |
| SMOG | Deep Seek vs Wenxin Yiyan | Wilcoxon signed-rank | 0.575 | 1.000 | No |
| SMOG | Doubao vs GPT-5 | Wilcoxon signed-rank | <.001 | 0.003 | Yes |
| SMOG | Doubao vs Tongyi Qianwen | Wilcoxon signed-rank | <.001 | 0.002 | Yes |
| SMOG | Doubao vs Wenxin Yiyan | Wilcoxon signed-rank | 0.147 | 1.000 | No |
| SMOG | GPT-5 vs Tongyi Qianwen | Wilcoxon signed-rank | 0.005 | 0.045 | No |
| SMOG | GPT-5 vs Wenxin Yiyan | Wilcoxon signed-rank | 0.025 | 0.251 | No |
| SMOG | Tongyi Qianwen vs Wenxin Yiyan | Wilcoxon signed-rank | <.001 | 0.004 | Yes |
| LW | Deep Seek vs Doubao | Paired t-test | 0.430 | 1.000 | No |
| LW | Deep Seek vs GPT-5 | Paired t-test | 0.013 | 0.127 | No |
| LW | Deep Seek vs Tongyi Qianwen | Paired t-test | 0.002 | 0.022 | No |
| LW | Deep Seek vs Wenxin Yiyan | Paired t-test | 0.572 | 1.000 | No |
| LW | Doubao vs GPT-5 | Paired t-test | <.001 | <.001 | Yes |
| LW | Doubao vs Tongyi Qianwen | Paired t-test | <.001 | <.001 | Yes |
| LW | Doubao vs Wenxin Yiyan | Paired t-test | 0.882 | 1.000 | No |
| LW | GPT-5 vs Tongyi Qianwen | Paired t-test | 0.063 | 0.625 | No |
| LW | GPT-5 vs Wenxin Yiyan | Paired t-test | 0.004 | 0.038 | No |
| LW | Tongyi Qianwen vs Wenxin Yiyan | Paired t-test | <.001 | 0.001 | Yes |
| C-PEMAT score | Deep Seek vs Doubao | Wilcoxon signed-rank | 0.299 | 1.000 | No |
| C-PEMAT score | Deep Seek vs GPT-5 | Wilcoxon signed-rank | <.001 | 0.007 | No |
| C-PEMAT score | Deep Seek vs Tongyi Qianwen | Wilcoxon signed-rank | 0.505 | 1.000 | No |
| C-PEMAT score | Deep Seek vs Wenxin Yiyan | Wilcoxon signed-rank | 0.606 | 1.000 | No |
| C-PEMAT score | Doubao vs GPT-5 | Wilcoxon signed-rank | 0.005 | 0.051 | No |
| C-PEMAT score | Doubao vs Tongyi Qianwen | Wilcoxon signed-rank | 0.858 | 1.000 | No |
| C-PEMAT score | Doubao vs Wenxin Yiyan | Wilcoxon signed-rank | 0.653 | 1.000 | No |
| C-PEMAT score | GPT-5 vs Tongyi Qianwen | Wilcoxon signed-rank | 0.007 | 0.066 | No |
| C-PEMAT score | GPT-5 vs Wenxin Yiyan | Wilcoxon signed-rank | <.001 | 0.005 | Yes |
| C-PEMAT score | Tongyi Qianwen vs Wenxin Yiyan | Wilcoxon signed-rank | 0.947 | 1.000 | No |
| GQS score | Deep Seek vs Doubao | Wilcoxon signed-rank | 0.675 | 1.000 | No |
| GQS score | Deep Seek vs GPT-5 | Wilcoxon signed-rank | 0.001 | 0.013 | No |
| GQS score | Deep Seek vs Tongyi Qianwen | Wilcoxon signed-rank | 0.020 | 0.195 | No |
| GQS score | Deep Seek vs Wenxin Yiyan | Wilcoxon signed-rank | 0.003 | 0.034 | No |
| GQS score | Doubao vs GPT-5 | Wilcoxon signed-rank | 0.002 | 0.021 | No |
| GQS score | Doubao vs Tongyi Qianwen | Wilcoxon signed-rank | 0.021 | 0.211 | No |
| GQS score | Doubao vs Wenxin Yiyan | Wilcoxon signed-rank | 0.008 | 0.083 | No |
| GQS score | GPT-5 vs Tongyi Qianwen | Wilcoxon signed-rank | <.001 | 0.002 | Yes |
| GQS score | GPT-5 vs Wenxin Yiyan | Wilcoxon signed-rank | <.001 | 0.001 | Yes |
| GQS score | Tongyi Qianwen vs Wenxin Yiyan | Wilcoxon signed-rank | 0.942 | 1.000 | No |

Note: A total of C (5,2) = 10 pairwise comparisons were performed per variable. Normally distributed variables were analyzed using paired t-tests; non-normally distributed variables were analyzed using Wilcoxon signed-rank tests. Adjusted P values were calculated using the Bonferroni correction (raw P × 10). Statistical significance was defined as adjusted P < 0.005.

**Supplementary Table 3.**

**Fixed effects tests from linear mixed-effects models with Question ID as random intercept**

| Variables | Platform F | Platform df | | Platform P | Dimension F | Dimension df | Dimension P |
| --- | --- | --- | --- | --- | --- | --- | --- |
| ARI | F=22.29 | | 4,76 | <.001 | F=3.36 | 4,15 | 0.037 |
| FRES | F=13.96 | | 4,76 | <.001 | F=4.80 | 4,15 | 0.011 |
| GFOG | F=26.15 | | 4,76 | <.001 | F=6.04 | 4,15 | 0.004 |
| FKGL | F=24.81 | | 4,76 | <.001 | F=5.43 | 4,15 | 0.007 |
| CL | F=37.07 | | 4,76 | <.001 | F=4.26 | 4,15 | 0.017 |
| SMOG | F=14.18 | | 4,76 | <.001 | F=3.43 | 4,15 | 0.035 |
| LW | F=10.85 | | 4,76 | <.001 | F=4.19 | 4,15 | 0.018 |
| C-PEMAT score | F=5.87 | | 4,76 | <.001 | F=1.22 | 4,15 | 0.344 |
| GQS score | F=19.63 | | 4,76 | <.001 | F=1.33 | 4,15 | 0.303 |

Note: All models included Question ID (20 questions) as a random intercept to account for the correlation among responses generated by different models for the same question. Fixed effects were tested using Type III tests with REML estimation and Satterthwaite approximation for denominator degrees of freedom.
